# Half-turned truncal switch operation after single ventricle palliation in a patient with borderline left heart hypoplasia

**DOI:** 10.1186/s13019-020-01357-y

**Published:** 2020-10-09

**Authors:** Tak-Hyuk Oh, Hanna Jung, Joon Yong Cho, Youngok Lee

**Affiliations:** Department of Thoracic and Cardiovascular Surgery, Kyungpook National University Hospital, Kyungpook National University School of Medicine, 130 Dongdeok-ro, Jung-gu, Daegu, 41944 South Korea

**Keywords:** Biventricular repair, Half-turned truncal switch operation, Left heart hypoplasia, Single ventricle palliation, Transposition of great arteries

## Abstract

**Background:**

The optimal surgical strategy for the correction of double outlet right ventricle (DORV, transposition of the great arteries [TGA] type) or TGA with ventricular septal defect (VSD), pulmonary stenosis (PS), and borderline small left ventricle (LV) is still controversial. The half-turned truncal switch operation (HTTSO) introduced by Yamagishi and colleagues is a good option, but it is still challenging in a patient with borderline small LV. We aimed to describe our experience of a case of HTTSO conversion from single ventricle palliation.

**Case presentation:**

A 5-year-old girl with single ventricle physiology was referred to our hospital from Kazakhstan for a Fontan operation. At the time of birth, she was diagnosed with DORV (TGA type), PS, and situs inversus totalis, with moderate valvar and subvalvar stenosis and a relatively small LV cavity. Her LV volume was not adequate to support the systemic circulation; therefore, doctors in Kazakhstan selected the single ventricle palliation course of treatment for the infant. At 4 months of age, she underwent left-sided modified Blalock-Taussig shunt, patent ductus arteriosus ligation, and atrial septectomy. At 2 years of age, shunt takedown, left bidirectional cavopulmonary shunt, and main pulmonary artery division were performed. Annual echocardiography of the patient showed that the LV size was growing too adequately to persist with the single ventricle palliation course of treatment. Via a multidisciplinary approach, we considered her LV to be suitable for biventricular repair and HTTSO was planned. The operation and postoperative course were uneventful. The patient was discharged on postoperative day 6 and went back to Kazakhstan.

**Conclusions:**

Based on our successful surgical outcome, in patients diagnosed with DORV (TGA type) or TGA with VSD, PS, and borderline LV, HTTSO after achieving adequate LV growth by single ventricle palliation may be considered a good alternative to conventional operations in patients at a high risk for initial biventricular repair.

## Background

The half-turned truncal switch operation (HTTSO) was first reported by Yamagishi and colleagues in 2003 as an alternative to the Rastelli, the Lecompte, and the Nikaidoh procedures for patients with double outlet right ventricle (DORV, transposition of the great arteries [TGA] type) or TGA with ventricular septal defect (VSD), and pulmonary stenosis (PS) [1, 2]. The advantages of the HTTSO are avoidance of late left ventricular outflow tract (LVOT) obstruction or right ventricular outflow tract (RVOT) obstruction and stretch or distortion of the coronary arteries.

Although the HTTSO is still challenging in patients with borderline small left ventricle (LV), it could be a treatment of choice after single ventricle palliation with adequate LV growth. We aimed to describe our experience of a case of HTTSO conversion from single ventricle palliation.

## Case presentation

A 5-year-old girl weighing 17.4 kg who had single ventricle physiology was referred to our hospital from Kazakhstan for a Fontan operation. She was diagnosed in her country with DORV (TGA type), PS, and situs inversus totalis. At the time of her birth, her pulmonary valve and LV were not adequate to support the systemic circulation because of moderate valvar and subvalvar stenosis and a relatively small LV cavity. Therefore, the doctors in Kazakhstan selected the single ventricle palliation course of treatment for the infant. At 4 months of age, she underwent a left-sided modified Blalock-Taussig shunt, patent ductus arteriosus ligation, and atrial septectomy performed by a local medical team in Kazakhstan. At 2 years of age, shunt takedown, left bidirectional cavopulmonary shunt (BCPS), and main pulmonary artery (PA) division were performed in Kazakhstan by our medical team volunteers who visited Kazakhstan every year to follow up on the patient. Annual echocardiography of the patient showed that the LV size was growing too adequately to persist with the single ventricle palliation course of treatment. We thought that the patient might be able to undergo biventricular repair, but precise examinations, such as computed tomography angiography with 3-dimensional reconstruction, and a multidisciplinary approach were required. We decided to invite her to our hospital in South Korea and formulated the next management plan for her.

**Fig. 1:**
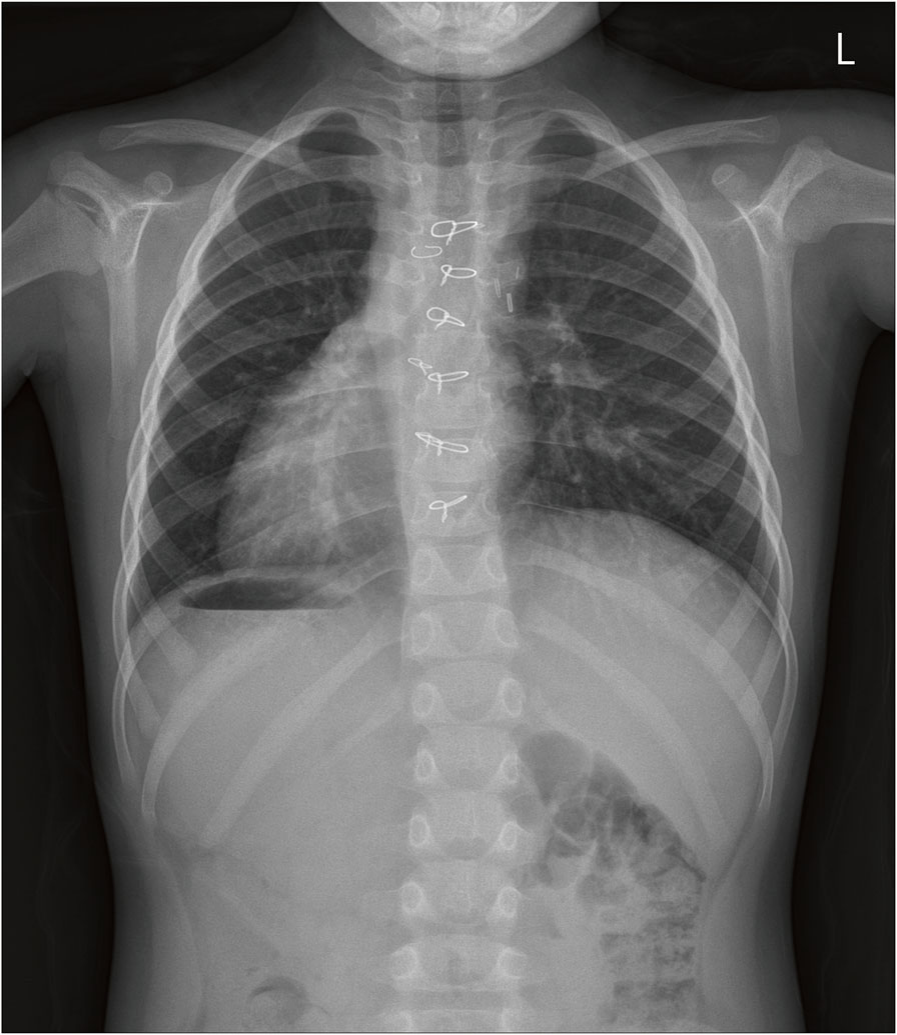
Preoperative chest radiography showing situs inversus totalis with dextrocardia

**Fig. 2:**
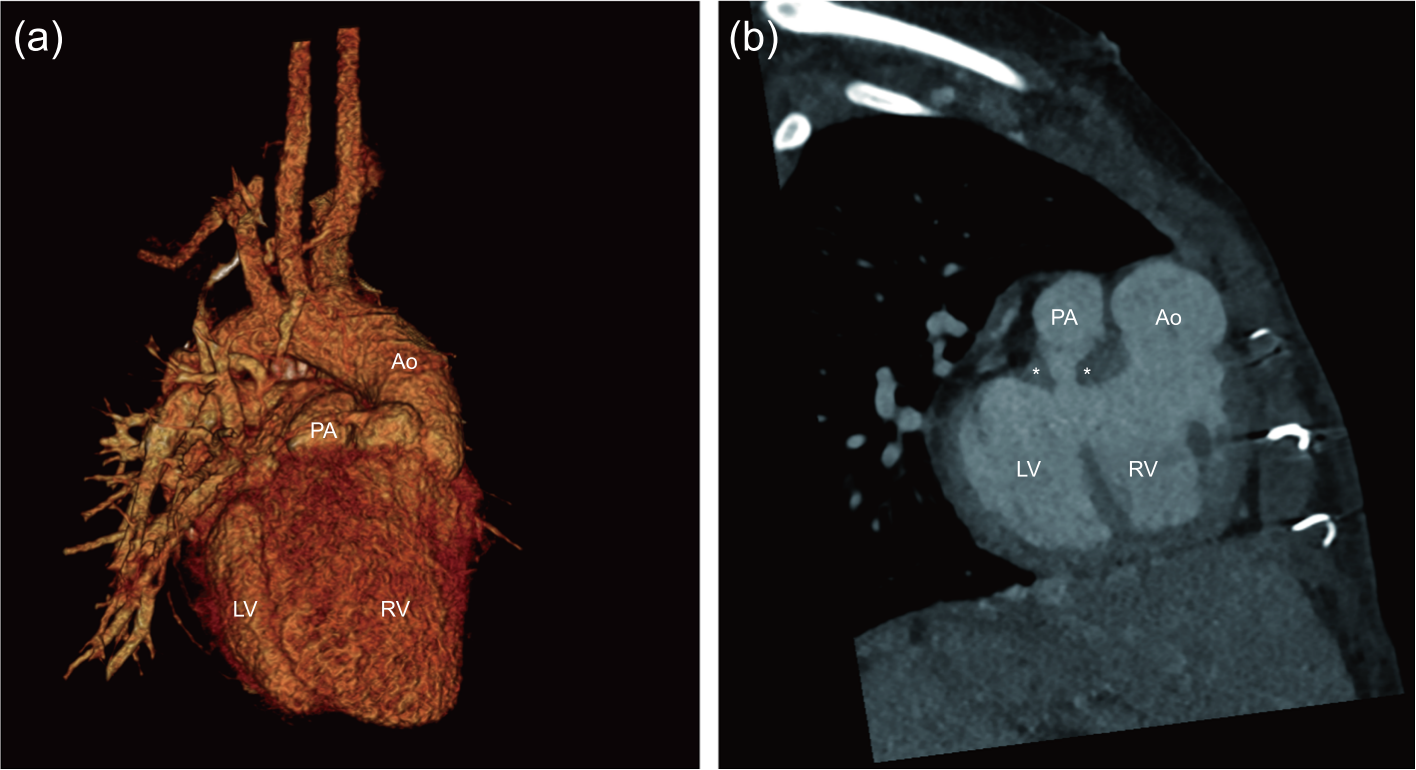
Preoperative chest computed tomography with (**a**) 3-dimensional reconstruction and (**b**) sagittal view. Double outlet right ventricle, subpulmonic muscular hypertrophy (white star), valvar pulmonary stenosis, and a relatively small left ventricle could be observed. Ao, ascending aorta; PA, pulmonary artery; LV, left ventricle; RV right ventricle

Chest radiography showed situs inversus totalis with dextrocardia (Fig. 1). Echocardiography demonstrated DORV (TGA type) with an anterior aorta, a large VSD, mild tricuspid regurgitation, and laminar flow from the superior vena cava (SVC) to the PA. The indexed LV end-systolic and end-diastolic volumes were 5.7 and 11.6 ml/m^2^, respectively. The mitral valve Z-score was − 1.76, and the mitral valve/tricuspid valve ratio was 0.87. The LV ejection fraction obtained using the Simpson method was 59.2%. A computed tomography angiography scan showed the same anatomical results with echocardiography (Fig. 2). Moreover, an inferior vena cava interruption with azygous continuation and hepatic veins draining directly into the right atrium was observed. Via a multidisciplinary approach, we considered her LV to be suitable for biventricular repair; thus, HTTSO was planned.

The operation was performed under routine cardiopulmonary bypass with the use of a Bretschneider histidine-tryptophan-ketoglutarate crystalloid solution, bicaval cannulation, and mild hypothermia induction. The technique for the HTTSO was similar to that described in our previous report [3]. In brief, the aorta was transected 10 mm above the coronary arteries and Yacoub type A coronary arteries were harvested. When the main PA stump was opened, the pulmonary valve was found to be bicuspid, and the valve leaflets appeared dysplastic, edematous, and thick. After truncal block resection, the VSD was closed using a bovine pericardial patch with interrupted 6–0 polypropylene double pledget supported sutures. The truncal block was half turned horizontally, and the posteriorly translocated aortic valve was anastomosed to the LVOT with 6–0 polypropylene continuous sutures. The coronary buttons were anastomosed to the corresponding aortic wall defects. After the takedown of BCPS, the PA was closed with a bovine pericardial patch. The posterior wall of the proximal PA stump was directly anastomosed to the posterior wall of the PA bifurcation without a Lecompte maneuver. The anterior wall of the neo-RVOT was covered by the bovine pericardial patch with the native valve leaflets. A longitudinal incision was made along the anterior wall of the cephalic end of the SVC to the innominate vein–SVC junction and then anastomosed directly to the right atrial auricle (RAA). This anastomosis was completed with a 7–0 polypropylene running suture, starting at the posterior wall with primary SVC-RAA approximation and anterior patch augmentation with bovine pericardium. Weaning from cardiopulmonary bypass was smoothly performed with the use of a low-dose prophylactic inotropic agent. The duration of the cardiopulmonary bypass and aortic cross clamp were 295 and 185 min, respectively.

**Fig. 3:**
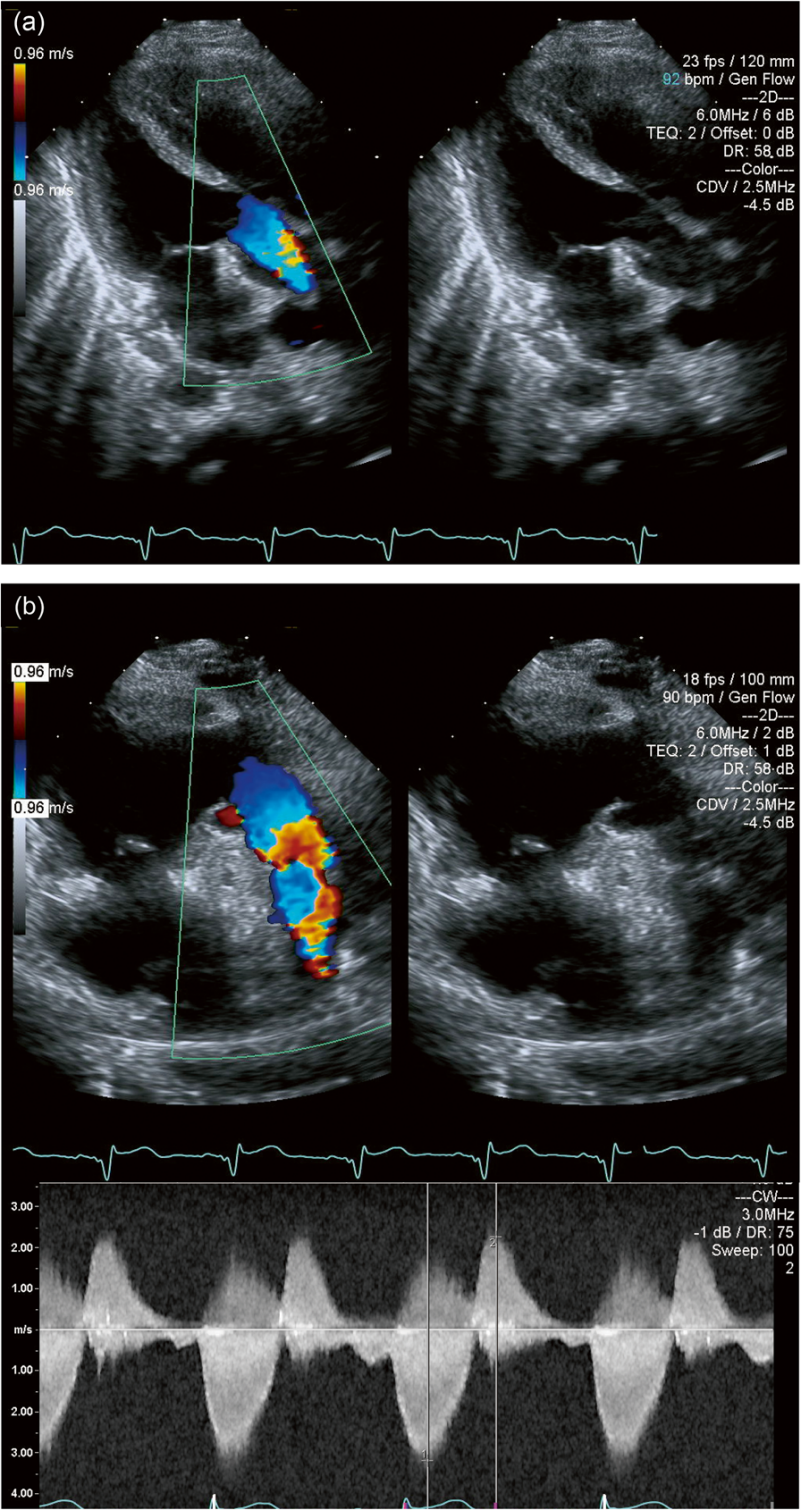
Postoperative echocardiography showing (**a**) a straight flow of the left ventricular outflow tract without stenosis and (**b**) mild pulmonary stenosis in the right ventricular outflow tract; peak velocity is 3 m/s

The patient was extubated at 1 day after the operation, and the postoperative course was uneventful. The patient was discharged on postoperative day 6 and went back to Kazakhstan. Postoperative echocardiography (Fig. 3) showed a normal LV function, straight flow of the LVOT without stenosis, mild tricuspid regurgitation, no mitral regurgitation, mild pulmonary regurgitation, and mild PS (peak velocity, 3 m/s). She had a normal sinus rhythm and had no stenosis at the SVC-RAA anastomotic site.

## Discussion and conclusions

The optimal approach for the correction of DORV (TGA type) or TGA with VSD, and PS is still controversial. This is especially true if the LV volume is not adequate to support the systemic circulation during the neonatal period.

Patients with mild LV hypoplasia may be amenable to eventual biventricular repair; however, they are at a higher risk if such a strategy is followed, particularly in infancy. Moreover, single ventricle palliation in a patient with two ventricles that are potentially adequate for biventricular repair can unnecessarily subject the patient to comorbidities related to the Fontan physiology [4, 5]. Emani et al. reported that in patients with borderline LV who underwent single ventricle palliation, it is possible to increase the LV dimension and the eventual biventricular conversion procedure by staged LV recruitment strategy. The rationale of the LV recruitment is to relieve inflow and outflow tract obstructions, promote blood flow through the LV, and simulate flow- and load-mediated growth [6, 7]. Li et al. recommended the re-evaluation of patients with previous BCPS and should not be considered as a one-way path to Fontan. First, due to the limitation of echocardiography, some patients with borderline LV were judged to be hypoplastic with under-filling and squashed by the overloaded right ventricle. Second, the LV size might be modified by hemodynamic factors caused by BCPS [8].

The common concept of BCPS is to relieve the source of volume and/or pressure load on the LV, and the ability of the LV to sustain a systemic function begins to decrease. However, since the volume and/or pressure load on the LV is not completely eliminated, the remaining blood flow might be able to mediate the growth of borderline LV with or without the LV recruitment strategy. Initially, in our patient, training the LV was not part of the treatment plan, but it was unintentionally trained or rehabilitated during the treatment course. The patient had relatively a large VSD, and BCPS as single ventricle palliation might have modified the LV ability. It gave us an excellent experience and awareness to consider biventricular repair of borderline LV in patients with single ventricle palliation.

The conventional approach to anatomic surgical repair in patients with DORV (TGA type) or TGA with VSD, and PS is the Rastelli or Lecompte procedure. However, these procedures have several serious drawbacks related to an earlier need for reinterventions or reoperation of the RVOT and a higher incidence of subaortic tunnel obstruction [1, 9]. To overcome these drawbacks, Nikaidoh reported a successful anatomic repair of the aortic root translocation without individual coronary artery transfer and biventricular outflow reconstruction for these patients in 1984, and thereafter, other modified operative methods of the aortic root translocation have been proposed [10]. However, the Nikaidoh operation carries a high risk of stretch or distortion of the coronary arteries in patients with a relatively large pulmonary annulus because the distance of the posterior transition of the aortic root depends on the size of the pulmonary annular diameter [1]. The HTTSO was designed for straight and non-obstructive LVOT and RVOT by using an autologous half-turned truncal block that involves both aortic and pulmonary valves. Therefore, the HTTSO overcomes various drawbacks of conventional procedures and offers the benefits of the Nikaidoh operation.

In patients diagnosed with DORV (TGA type) or TGA with VSD, PS, and borderline LV, HTTSO after LV growth by single ventricle palliation is a good alternative to conventional operations in patients at a high risk for initial biventricular repair.

## Data Availability

Not applicable.

## Abbreviations

HTTSO: Half-turned truncal switch operation
TGA: Transposition of the great arteries
DORA: Double outlet right ventricle
VSD: Ventricular septal defect
PS: Pulmonary stenosis
LVOT: Left ventricular outflow tract
RVOT: Right ventricular outflow tract
LV: Left ventricle
BCPS: Bidirectional cavopulmonary shunt
PA: Pulmonary artery
SVC: Superior vena cava
RAA: Right atrial auricle
